# Research on Green Adsorbents

**DOI:** 10.3390/molecules29081855

**Published:** 2024-04-19

**Authors:** Małgorzata Wiśniewska, Piotr Nowicki

**Affiliations:** 1 Department of Radiochemistry and Environmental Chemistry, Faculty of Chemistry, Institute of Chemical Sciences, Maria Curie-Sklodowska University, M. Curie-Sklodowska Sq. 3, 20-031 Lublin, Poland; 2 Department of Applied Chemistry, Faculty of Chemistry, Adam Mickiewicz University, Uniwersytetu Poznańskiego 8, 61-614 Poznań, Poland

## 1. Introduction

Adsorption processes play a crucial role in air purification, wastewater treatment, soil remediation technologies, noble metals recovery, and long-term energy storage systems [1,2,3]. Among the many advantages of using this phenomenon, the following deserve special emphasis: simplicity of operation, easy control of its course, high efficiency and selectivity, desirable catalytic properties, good reuse potential, favorable economic considerations, and the availability of a wide range of various adsorbents. In recent years, thanks to enormous technological progress, which also allows the integration of materials science, chemistry, biology, geology, chemical engineering, environmental science, and other disciplines, a variety of new adsorption materials with excellent physicochemical properties have been developed [4,5]. Due to the special emphasis on the introduction of green technologies in accordance with the principles of sustainable development, this trend is also developing intensively in the field of adsorption materials [6,7,8]. Green or environmentally friendly adsorbents are derived from minerals, natural polymers, plants, and agricultural crop sources, which ensures a very low production cost while maintaining competitive adsorption parameters compared to classic adsorbents [9,10,11,12]. The term “green” refers not only to the management of biomass that is troublesome to store but also to the application of green fabrication methods (using small amounts of less aggressive chemical reagents), the multiple and simple regeneration of waste adsorbents, as well as its utilization in natural processes (excellent biodegradability) [13,14,15]. Numerous green adsorbent materials are presently synthesized, which demonstrates the economic and ecological feasibility of the large-scale application of eco-friendly operations and strategies of removal for different categories of environmental pollutants and developing new materials for heat storage [16,17,18].

This Special Issue contains reviews and research papers dedicated to the presentation of the latest achievements in the field of synthesis, characterization, and the application of adsorbents from the “green” group. A brief overview of the included manuscripts is shown below, and we hope that it will encourage potential readers to explore them in more detail.

## 2. An Overview of Published Articles

Juraj Bujdák’s review article (contribution 1) deals with the most serious and problematic issues found in the literature on the adsorption of dyes on clay minerals. They include the insufficient characterization of adsorbents, the influence of impurities on the adsorption of dyes, and the choice of inappropriate models for the description of very complex clay mineral systems. This paper discusses the main processes accompanying adsorption in colloidal systems of clay minerals. In turn, this critical review proposes valuable tips to avoid some faults in the characterization of the adsorption of organic dyes on clay minerals, improve the procedure for determining the adsorbed amount, evaluate the results correctly, and find an appropriate theoretical interpretation.

The article by Victoria Paientko et al. (contribution 2) focuses on the textural and electrochemical properties of nanocomposite fillers based on the mixture of rose clay/hydroxyapatite/nanosilica enriched with acai leaf powder. The authors proved that the sample preparation method affects the particle size (aggregation tendency), surface charge density, zeta potential, and pH value of the aqueous suspensions. Moreover, the release of cyanidin-3,5-diglycoside (anthocyanin) as a bioactive compound is greater for the composites than for acai with rose clay alone. Thus, composites with clay/nanosilica/vegetable raw materials/hydroxyapatite can be considered as better systems for cosmetics and medicinal preparations than rose clay without additives.

Contribution 3, authored by Barbara Charmas et al., presents the preparation of activated carbons from wheat bran as a result of pyrolysis and physical activation (carbon dioxide or/and steam) with additional modification with superheated steam using microwave radiation. The obtained materials were characterized mainly by their microporous nature, and the additional modification resulted in the extension of the already existing pores. The activated carbons showed an amphoteric character with a predominance of acidic groups. They were characterized by an amorphous structure and were stable up to the temperatures of 450–550 °C. It was also shown that they have very good sorption capacity towards methylene blue.

The research presented by Agnieszka Szymaszek-Wawryca et al. (contribution 4) concerns the synthesis of two series of MCM-36 zeolites intercalated with various pillars and modified with iron, as well as their analysis with respect to physicochemical properties and catalytic performance for the NH_3_-SCR process. It was found that the characteristic MWW morphology of MCM-36 can be obtained successfully using silica, alumina, and iron oxide as pillars. Additionally, the one-pot synthesis of the material with iron resulted in the incorporation of monomeric Fe^3+^ species into the framework positions. The results of catalytic tests revealed that the one-pot-synthesized sample intercalated with silica and alumina was the most efficient catalyst of NO reduction, exhibiting ca. 100% activity at 250 °C.

Minh Hoang Nguyen et al. (contribution 5) examined the possibility of using biochar-based composite sorbents to develop new, promising, and efficient heat storage materials. Biochar-based composites with defined salt loadings (5, 10, 15, and 20%) were produced by the MgSO_4_ impregnation of the biochar matrix derived from corn cobs (CC). The new materials demonstrated the high-water sorption capacity. The cyclability of the MgSO_4_/biochar composites was confirmed through six hydration/dehydration cycles, demonstrating the stability and durability of the 20MgCC composite (for which the energy storage density was 635 J/g). The authors concluded that obtained composites open a low-carbon path for the production of sustainable thermal energy storage materials and their applications.

The plant waste-derived pelletized biochar obtained from different feedstock and pyrolyzed at 600 °C was applied for the adsorption of nitazoxanide (an antiparasitic drug for SARS-CoV-2 treatment) by Artur Sokołowski et al. (contribution 6). It was demonstrated that the drug’s adsorption was fast, enabling its efficient removal after one hour. The highest adsorption capacity was noted for biochar obtained from biogas production (14 mg/g). The presence of inorganic ions (Cl^−^, NO_3_^−^) had an unfavorable effect on adsorption (a decrease in the adsorption level of 20–30%), and carbonyl surface groups of carbon materials were the most effective in hindering this process (with an adsorption decrease by about 60%). The environmentally relevant concentration of dissolved organic matter (DOM) at the level of 10 mg/L did not affect drug removal.

In the seventh contribution submitted by Hubicki et al., the natural sorbent Nitrolite impregnated with Aliquat 336 using a new warm impregnation method was obtained. This material was applied for the sorption of noble metals, such as palladium (II), platinum (IV), and gold (III), from the model chloride solutions. The values of the sorption capacity for these metal ions were 47.63, 51.39, and 73.43 mg/g, respectively. Additionally, the impregnated sorbent Nitrolite–Aliquat 336 was used in the investigations of the sorption of platinum (IV), palladium (II), and rhodium (III) ions from a real solution. The applied sorbent proved to be suitable for the recovery of platinum (IV) and palladium (II) ions, whereas rhodium ions were not bounded from the leached solutions. Moreover, the desorption (using 1 M of thiourea in the 1 M HCl solution) was above 94% for all noble metals.

Agnieszka Ślosarczyk et al. (contribution 8) designed an eco-efficient composite based on alkali-activated materials (AAMs) modified with titanium dioxide and exhibiting self-cleaning properties for sustainable construction. Significant emphasis was placed on determining the role of the precursor type, the amount of sodium silicate, and the addition of titanium dioxide on the rheological and mechanical properties of AAMs. Blast furnace slag (BFS) was used as the source material, which was then substituted in subsequent formulations with metakaolinite at 50%, as well as fly ash and zeolite at 30%. The most activated AAMs, in which blast furnace slag and its mixture with metakaolinite were used as precursors, achieved compressive strengths of 50 MPa. The obtained results indicated that TiO_2_ addition improved the final strengths and slightly lowered the heat of hydration and spreading of the AAM mortars. The best self-cleaning properties were achieved by composites containing furnace slag, fly ash, and 2% titanium dioxide.

The toxicity of two pesticides, diazinon (DAZ) and atrazine (ATR), before and after montmorillonite-catalyzed ozonation on duckweed *Lemna minor* was comparatively investigated by Amina Benghaffour et al. (contribution 9). The results demonstrate the role of clay-containing media in the evolution at the time of pesticide’s negative impact on *L. minor* plants. The conversion of pesticides exceeded 94% after 30 min of ozonation in the presence of both Na^+^- and Fe^2+^-exchanged montmorillonites. It was shown that plant growth inhibition involved excessive oxidative stress depending on the pesticide concentration, molecular structure, and degradation degree. Pesticide adsorption and/or conversion by ozonation on clay surfaces significantly reduced the toxicity towards *L. minor* plants, particularly in the presence of Fe(II)-exchanged montmorillonite. These findings can be helpful in monitoring pesticide ecotoxicity according to clay-containing host media and the exposure time to ambient factors.

The research presented by Dorota Paluch et al. (contribution 10) investigates the possibility of removing methylene blue and methyl red from aqueous solutions using activated carbons obtained by the chemical activation (using Na_2_CO_3_, K_2_CO_3_, and H_3_PO_4_) of caraway seed. The specific surface area of the obtained activated carbons ranged from 269 to 926 m^2^/g, and the iodine numbers for these samples ranged from 278 to 1107 mg/g. Dose effect studies have shown that with increasing sample mass, the sorption capacity of the adsorbent decreases, whereas the percentage removal of dye contaminants from the aqueous solution increases. It was also found that the increase in the pH value causes an increase in the sorption of methylene blue on the activated carbons and a decrease in the amount of adsorbed methyl red. Finally, the presented results proved that chemical activation with potassium carbonate leads to obtaining a carbonaceous adsorbent with the most favorable textural parameters and the highest sorption capacity toward the dye pollutants.

Anjiu Zhao et al. (contribution 11) used zeolitic imidazolate frameworks (ZIFs) as an efficient adsorbent of organic pollutants. The ZIF-8 was successfully loaded onto lignocellulose (LC) to further produce ZnO/LC using the in situ growth method and hydrothermal treatment. In the next step, Fe_3_O_4_ nanoparticles (Fe_3_O_4_ NPs) were loaded onto ZnO/LC to prepare the magnetic Fe_3_O_4_/ZnO/LC adsorbent for removing tetracycline (TC) and congo red (CR) pollutants from the aqueous solution. The adsorbent dosage, solution pH, contact time, temperature, and initial adsorbate concentration effects were investigated. The adsorption capacities of TC and CR reached the maximum values of 383.4 and 409.1 mg/g, respectively. The authors proved that this novel adsorbent could be rapidly separated from the aqueous solution, suggesting its high potential to remove pollutants in wastewater.

The twelfth manuscript in this Special Issue was prepared by Karolina Ptaszyńska et al., and it deals with the sustainable and selective synthesis of glycerol carbonate (GC) from urea and glycerol under ambient pressure using carbon-fiber-supported (by Zn, Ba, Cr, Mg) metal oxide catalysts. Carbon fibers (CFs) were prepared via the catalytic chemical vapor deposition method (CCVD) using Ni as a catalyst and liquefied petroleum gas (LPG) as a cheap carbon source. The obtained catalysts (10% MexOy/CFox) were tested in the reaction of urea glycerolysis at 140 °C for 6 h under atmospheric pressure, using an equimolar ratio of reagents and an inert gas flow for NH_3_ removal. It was demonstrated that all of the prepared catalysts increased the glycerol conversion and glycerol carbonate yield compared to the blank test, and the best catalytic performance was shown by the CFox-supported ZnO and MgO systems. Under the best reaction conditions used, the high yield of glycerol carbonate of about 40%, together with ~100% selectivity to GC, was obtained over the ZnO/CFox catalyst.

Robert Wolski et al. (contribution 13) obtained carbon adsorbents via the physical activation of the corn digest with CO_2_ and characterized the textural and acidic-basic properties of these materials. In addition, their sorption capacities towards methylene blue and methyl red, as well as the kinetics of the dye adsorption process, were determined. The carbonaceous adsorbents were characterized by a medium-developed specific surface area (320–616 m^2^/g). The sorption capacity of these materials against methylene blue ranged from 40 to 146 mg/g, and methyl red covered the range from 31 to 113 mg/g. Moreover, adsorption studies showed that as the process temperature increased, the removal efficiency of methylene blue and methyl red became more effective. It has been proved that applying the adsorption of dyes on activated carbons is endothermic and spontaneous, as well as the fact that its mechanism is primarily based on chemisorption.

A new phosphorus-containing sorbent prepared by the copolymerization of ethylene glycol dimethacrylate (EGDMA) and trimethylvinyl silane (TMVS) with diphenylvinylphoshine oxide (DPVO) was obtained by Monika Wawrzkiewicz et al. (contribution 14). It was fully characterized and applied in the removal of cationic dyes such as C.I. Basic Yellow 2 (BY2), C.I. Basic Blue 3 (BB3), and C.I. Basic Red 46 (BR46) using the batch method. Spectroscopic analysis indicated that the phosphinoyl group was introduced into the sorbent structure. Equilibrium adsorption data were fitted to the Langmuir, Freundlich, Temkin, and Dubinin–Radushkevich isotherm models. The Freundlich model is the most suitable to describe the adsorption of BB3 and BY2, whereas the Langmuir model is the most adequate to describe the adsorption of BR46. The kinetics of the dye adsorption follows the assumptions of the pseudo-second-order. The presence of Na_2_SO_4_ and the cationic surfactant in the aqueous solutions inhibited dye retention using the DPVO–EGDMA–TMVS material. Adsorbent regeneration efficiency did not exceed 60% using 1 M NaCl and 1 M HCl solutions in the presence of 50% *v*/*v* methanol. The authors conclude that the obtained results for the removal of basic dyes using the DPVO–EGDMA–TMVS adsorbent are important both from a theoretical perspective and practical application; however, a detailed economic analysis the of process is required.

The last paper, authored by Wiśniewska et al. (contribution 15), is focused on the assessment of the usefulness of the low-quality brown coal and the ash obtained as a result of its combustion, as well as used gear oil for the production of mineral–carbon adsorbents. The obtained materials were characterized in terms of their textural parameters, the basic acidic character of the surface, the contribution of mineral matter to the structure, as well as their suitability for drinking water purification (methylene blue and methyl orange removal). The effect of the initial dye concentration, temperature, and pH of the system, as well as the phase contact time, were investigated. It was shown that obtained mineral–carbon composite and activated carbons significantly differed not only in terms of the elemental composition and chemical character of the surface (from slightly acidic to strongly alkaline) but also showed a very diverse degree of specific surface development (from 21 to 656 m^2^/g) and the type of porous structure generated (from micro/mesoporous to typically mesoporous). The most effective adsorbent in relation to methylene blue turned out to be the sample obtained as a result of the chemical activation of brown coal, which was able to adsorb 233.4 mg/g of this dye. In the case of methyl orange removal, both carbon materials (chemically and physically activated) showed similar adsorption capacities at the level of approx. 85 mg/g.

## 3. Conclusions

This collection of articles devoted to green adsorbents indicates many areas of their applications. The main trend in the presented papers seems to be the synthesis of activated carbons from biomass and their use to remove pollutants from the aqueous solution (mostly synthetic dyes with different ionic characters). In addition to waste plant raw materials (such as wheat bran, corn cobs, and caraway seed), low-quality brown coal, ash from its combustion, and used gear oil were involved as precursors for carbonaceous materials production. This approach decreases the amount of hazardous and toxic waste and also leads to a reduction in greenhouse gas emissions resulting from biomass processing and the binding of elemental carbon into a stable structure by pyrolysis. Another direction of research is the development of green adsorbents based on zeolites and carbon fibers that meet the high requirements for catalytic processes related to air purification from hazardous gases, such as ammonia and nitrogen oxides. Other important trends of green synthesis application are the processes of noble metals recovery, energy storage, production of cosmetics, as well as pesticide and drug toxicity reduction towards living organisms in contaminated soil and water.

Such a wide area of studies indicates the comprehensive possibilities of using green materials in various areas of human life. On the other hand, it reveals a constant search for novel, more effective solutions that are more and more environmentally friendly. Nowadays, scientists are reaching for new waste materials, which, on the one hand, pose a serious burden to ecosystems but, on the other hand, are valuable precursors to obtain modern adsorption systems with separation, catalytic, and energy potential. Very often, composite materials are designed because their interesting properties meet all the criteria required in a specific practical application. Such an advanced approach to the development of green adsorbents would not be possible without enormous and continuous progress in the field of advanced measurement equipment, which enables the comprehensive characterization of the obtained materials. Moreover, the multidirectional description of the tested systems allows for the determination of various mechanisms of the surface processes occurring at the phase boundaries, which, in turn, guarantees their effective implementation on a large technological scale.
